# Worldwide distribution and environmental origin of the Adelaide imipenemase (AIM-1), a potent carbapenemase in *Pseudomonas aeruginosa*

**DOI:** 10.1099/mgen.0.000715

**Published:** 2021-12-17

**Authors:** Anteneh Amsalu, Sylvia A. Sapula, Jonathan J. Whittall, Bradley J. Hart, Jan M. Bell, John Turnidge, Henrietta Venter

**Affiliations:** ^1^​ UniSA Clinical and Health Sciences, Health and Biomedical Innovation, University of South Australia, Adelaide, Australia; ^2^​ Department of Medical Microbiology, University of Gondar, Gondar, Ethiopia; ^3^​ Australian Centre for Antimicrobial Ecology, The University of Adelaide, Adelaide, Australia; ^4^​ Adelaide Medical School, University of Adelaide, Adelaide, Australia

**Keywords:** Adelaide imipenemase (AIM-1), antimicrobial resistance, carbapenem resistance, *Pseudomonas aeruginosa*, *Pseudoxanthomonas mexicana*

## Abstract

Carbapenems are potent broad-spectrum β-lactam antibiotics reserved for the treatment of serious infections caused by multidrug-resistant bacteria such as *Pseudomonas aeruginosa *. The surge in *P. aeruginosa* resistant to carbapenems is an urgent threat, as very few treatment options remain. Resistance to carbapenems is predominantly due to the presence of carbapenemase enzymes. The assessment of 147 *P *. *aeruginosa* isolates revealed that 32 isolates were carbapenem non-wild-type. These isolates were screened for carbapenem resistance genes using PCR. One isolate from wastewater contained the Adelaide imipenemase gene (*bla*_AIM-1_) and was compared phenotypically with a highly carbapenem-resistant clinical isolate containing the *bla*_AIM-1_ gene. A further investigation of wastewater samples from various local healthcare and non-healthcare sources as well as river water, using probe-based qPCR, revealed the presence of the *bla*_AIM-1_ gene in all the samples analysed. The widespread occurrence of *bla*_AIM-1_ throughout Adelaide hinted at the possibility of more generally extensive spread of this gene than originally thought. A blast search revealed the presence of the *bla*_AIM-1_ gene in Asia, North America and Europe. To elucidate the identity of the organism(s) carrying the *bla*_AIM-1_ gene, shotgun metagenomic sequencing was conducted on three wastewater samples from different locations. Comparison of these nucleotide sequences with a whole-genome sequence of a *P. aeruginosa* isolate revealed that, unlike the genetic environment and arrangement in *P. aeruginosa *, the *bla*_AIM-1_ gene was not carried as part of any mobile genetic elements. A phylogenetic tree constructed with the deduced amino acid sequences of AIM-1 suggested that the potential origin of the *bla*_AIM-1_ gene in *P. aeruginosa* might be the non-pathogenic environmental organism, *Pseudoxanthomonas mexicana *.

## Data Summary

The authors confirm that all supporting data, code and protocols have been provided within the article or through supplementary data files.

**Impact Statement**

Carbapenem-resistant *Pseudomonas aeruginosa* is a critical priority pathogen in urgent need of new antimicrobial drug development according to the World Health Organization. An understanding of the molecular mechanisms driving carbapenem resistance in *P. aeruginosa,* as well as the origin and distribution of carbapenem resistance determinants, is thus crucial in tackling this pathogen. We identified the *bla*_AIM-1_ gene as a major determinant for high-level carbapenem resistance in *P. aeruginosa *. This gene codes for the AIM-1 carbapenemase, a potent beta-lactamase enzyme that is a thousand times more efficient in the hydrolysis of beta-lactam antibiotics than other common carbapenemases. Moreover, the *bla*_AIM-1_ gene was widely distributed in local wastewater samples and, significantly, also worldwide. Further investigation revealed that this gene originated from the non-pathogenic, ubiquitous environmental organism *Pseudoxanthomonas mexicana *. These findings indicate a worldwide distribution of the *bla*_AIM-1_ gene that can be mobilized and transferred to pathogens under optimal circumstances. Furthermore, the detection of AIM-1 in *P. aeruginosa* from other countries indicates that pathogens carrying this gene are not geographically limited to one city.

## Introduction

Carbapenems are highly potent broad-spectrum β-lactam antibiotics reserved for the treatment of serious infections caused by extended-spectrum β-lactamase (ESBL)-producing and multidrug-resistant (MDR) Gram-negative bacteria [1–3]. The last two decades have seen an increase in the global dissemination of ESBL-producing Gram-negative bacteria [4, 5], which has led to an increase in the use of carbapenems to treat infections caused by these bacteria [6]. This has led to the emergence of carbapenem-resistant bacteria [7], which is exemplified by the emergence of carbapenem-resistant *P. aeruginosa *, which is now listed by the World Health Organization (WHO) as a critical priority pathogen in need of research and new drug development [8]. Infections caused by carbapenem-resistant *P. aeruginosa* are the leading cause of mortality in critically ill, immunocompromised, surgical and burn wound patients [9–11]. Resistance to carbapenems in *P. aeruginosa* can occur through three major mechanisms: acquisition of carbapenemases (carbapenem-hydrolyzing enzymes), which hydrolyze the β-lactam ring directly; overexpression of drug efflux pumps, such as those belonging to the resistance–nodulation–division (RND) family, which facilitate the export of carbapenems from the cell; and reduction in cell permeability through the downregulation or loss of OprD porins required for carbapenem entry [12, 13]. Notably, the acquisition of carbapenemases is of great concern, as these can hydrolyze most β-lactam antibiotics and are encoded by genes carried on mobile genetic elements, which aid in the horizontal transfer of these genes to other bacterial species [6, 14, 15].

Carbapenemases belong to two large families, the serine and metallo-β-lactamases [16]. The serine β-lactamases include Ambler classes A and D β-lactamases and require a serine residue at the active site. Metallo-β-lactamases (MBLs) include the Ambler class B enzymes, which require one or two zinc ions for their catalytic activity [17]. Unlike serine β-lactamases, MBLs are not inhibited by commonly used clinical inhibitors, such as clavulanic acid, tazobactam, or sulbactam [18]. Their ability to hydrolyze almost all carbapenems efficiently [19, 20] and their rapid dissemination threatens current treatment options.

The widespread distribution of carbapenemases is largely due to their dissemination on mobile genetic elements such as plasmids, transposons and insertion sequences [14]. Such dissemination and subsequent acquisition are illustrated by the Adelaide imipenemase 1 (AIM-1), carried by *P. aeruginosa *. AIM-1 is a chromosomally encoded MBL enzyme that has been mobilized via an insertion sequence common region (ISCR) [21]. First detected in Adelaide, Australia in 2008 [21], kinetic analysis of the AIM-1 MBL enzyme revealed that it had a broad-spectrum hydrolysis of β-lactams, including carbapenems, but not aztreonam [21]. It is a thousand times more efficient in its ability to hydrolyze common carbapenems, such as meropenem and imipenem, compared to the most frequently occurring MBLs, such as VIM-2 (Verona integron-encoded MBL) and IMP (imipenemase) enzymes [14, 21]. Although both the crystal structure and the catalytic efficiency of this enzyme for different β-lactam antibiotics have been elucidated [21, 22], the origin, distribution and contribution to carbapenem resistance have not been established.

In this study, we undertook phenotypic and genotypic characterization of two *P. aeruginosa* isolates carrying the *bla*_AIM-1_ gene. One isolate (WCH6691) originated from a clinical specimen, while the other isolate (PA0545) was obtained from healthcare-associated wastewater assessed in our previous study [23]. Furthermore, we extended the search for the *bla*_AIM-1_ gene and screened both healthcare- and non-healthcare-associated wastewater, including water sourced from a river using a probe-based qPCR assay. In addition, metagenomic sequencing was performed on a select number of wastewater samples. Finally, by employing bioinformatic analysis on the sequencing data obtained in this study in combination with available public databases we predicted the origin of the *bla*_AIM-1_ gene.

## Methods

### Bacterial strains used

A total of 147 *P *. *aeruginosa* isolates were collected from human, animal and wastewater sources. The *P. aeruginosa* clinical isolate (WCH6691) was obtained from the Australian group on antimicrobial resistance (AGAR). Of these, 32 isolates were found to be resistant to the most frequently used carbapenems, imipenem or meropenem, and were investigated in this study.

### PCR screening of carbapenem resistance genes

All carbapenem-resistant *P. aeruginosa* isolates were screened with PCR for the *bla*_IMP_, *bla*_VIM_ *, bla*_NDM_ *, bla*_AIM_ *, bla*_KPC_ and *bla*_OXA_ genes using published primer sets [24] and genomic DNA was extracted from a single colony grown on a selective cetrimide *Pseudomonas* agar plate (Oxoid, Basingstoke, UK). The single colony was added to 100 µl molecular biology-grade water, which was then boiled at 98 °C for 10 min, cooled on ice and centrifuged at 4000 r.p.m. for 2 min. Finally, 90 µl of DNA containing supernatant was transferred into a sterile Eppendorf tube and stored at – 20 °C. Positive control strains for each resistance gene were obtained from the Australian Centre for Antimicrobial Resistance Ecology, Adelaide (Table 1).

**Table 1.** Carbapenemase-positive control strains used in this study

Control strain	Carbapenemases gene
D-034 *Klebsiella pneumoniae *	*bla*_KPC-2_
D-030 *K. pneumoniae *	*bla*_VIM-*1*_
D-037 *K. pneumoniae *	*bla*_OXA-48_
D-053 *K. pneumoniae *	*bla*_NDM-4_
D-021 *Serratia marcescens *	*bla*_IMP-1_
D-046 *Pseudomonas aeruginosa* *	*bla*_AIM-1_

*Submitted to the NCBI as WCH6691 and used throughout the paper.

AGAR, Australian group for antimicrobial resistance.

### Antimicrobial susceptibility testing

The minimum inhibitory concentrations (MICs) for the carbapenemase-positive *P. aeruginosa* isolates, WCH6691 and PA0545, were determined using the microdilution method and the results were interpreted according to 2020 European Committee on Antimicrobial Susceptibility Testing (EUCAST) (https://mic.eucast.org/) epidemiological cut-off (ECOFF) values. Strains with MICs at or below the ECOFF were defined as wild-type (WT) and those with MICs above the ECOFF were defined as non-wild-type. These isolates were tested against the following antimicrobial agents: ceftazidime, cefepime, tobramycin, ciprofloxacin, levofloxacin, imipenem, meropenem, doripenem, biapenem, colistin, piperacillin–tazobactam and trimethoprim–sulfamethoxazole. Since no ECOFF value has been established by EUCAST for biapenem against *P. aeruginosa *, an MIC >4 mg l^−1^ was considered to be non-wild-type according to the previous study [25]. *Escherichia coli* ATCC 25922 and *P. aeruginosa* ATCC 27853 were included in each experiment as quality control strains.

### DNA extraction and whole-genome sequencing

Genomic DNA from the *P. aeruginosa* isolates, WCH6691 and PA0545, carrying the *bla*_AIM-1_ gene were extracted using the MN NucleoSpinMicrobial DNA kit (Machery-Nagel GmbH and Co. KG, Duren, Germany) following the manufacturer’s instructions [23].

The library preparation and whole-genome sequencing (WGS) were performed at SA Pathology (Adelaide, SA, Australia) using the Nextera XT sample preparation kit on the Illumina NextSeq 550 platform (Illumina, Inc., San Diego, CA, USA).

### Sequence quality filtering, assembly and genome annotation

The 150 bp raw paired-end sequenced reads were analysed using the TORMES pipeline v.1.2 [26]. Briefly, the quality of reads was assessed using FastQC v.0.11.5. Adaptors and low-quality reads were removed using Trimmomatic v.36 [27] and taxonomy was classified using kraken2 [28]. Sequences were *de novo* assembled using SPAdes v 3.14.1 [29]. The genomes were annotated using prokka v1.14.5 (https://github.com/tseemann/prokka) [30] and the Rapid Annotation using Subsytems Technology (RAST) server v.2.0 [31]. Multilocus sequence type (MLST) were determined using the PubMLST *P. aeruginosa* typing schemes (https://pubmlst.org/) [32] and the mlst software (T. Seemann, https://github.com/tseemann/mlst). Antimicrobial resistance (AMR)-encoding genes were screened using Abricate against the Resfinder [33], Comprehensive Antibiotic Resistance Database (CARD) [34] and Antibiotic Resistance Gene-ANNOTation (ARG-ANNOT) [35] databases (T. Seemann, https://github.com/tseemann/abricate).

### Assessment of efflux pump activity using the checkerboard assay

To determine if RND efflux pumps may have contributed to meropenem and/or imipenem resistance, a checkerboard assay was performed as previously described [23]. Briefly, the MICs of imipenem- and/or meropenem-resistant isolates were determined in the presence of the efflux pump inhibitor, phenylalanine arginine β-naphthylamide (PAβN) (Sigma-Aldrich, St Louis, MI, USA) [36]. A decreased MIC value (≥4-fold) in the presence of PAβN suggests a contribution of RND efflux pumps in resistance to these antibiotics.

### RNA extraction

Total RNA was extracted from the mid-log-phase *P. aeruginosa* isolates, WCH6691 and PA0545, without and with imipenem shock (2× MIC, 30 min). RNA was extracted as described previously [23].

### Reverse-transcription quantitative polymerase chain reaction (RT-qPCR)

From imipenem-shocked and non-shocked cells, the relative level of mRNA expression in cells with/without was determined using a magnetic induction cycler instrument (Bio Molecular Systems, Adelaide, Australia) following KAPA SYBR FAST One-Step RT-qPCR master mix (2x) (Sigma-Aldrich, MI, USA) kit instructions [23]. The 2^−ΔΔcq^ values were normalized to that of the PAO1 control strain and relative expression of gene of interest was calculated using *rpsL* as an internal control [37]. The relative expression levels of the *bla*_AIM-1_ gene between isolates with/without imipenem were compared. Primers *bla*_AIM-1__F 5′-CTGAAGGTGTACGGAAACACCTG-3′ and *bla*_AIM-1_R_ 5′-GATGTTGGCCAGGATCTGTG −3′ and a previously described primer for *rspL* [23] were used.

### DNA extraction and detection of *bla*_AIM-1_ gene from wastewater using probe-based qPCR

Genomic DNA was extracted from composite (12 h) collections of wastewaters from four healthcare (HWW-1 to 4) and four non-healthcare (NHWW-1 to 4) settings. Non-healthcare settings included a retirement village, community wastewater and water sourced from a river. Samples of 100 ml were initially filtered through an 8 µm cellulose nitrate filter to remove debris and then through a 0.25 µm pore size membrane filter (both from Biotech GmbH, Göttingen, Germany). Genomic DNA was then extracted from the membranes using the DNeasy PowerWater kit (Qiagen, Hilden, Germany) following the manufacturer’s instructions. The presence of the *blaA*_IM-1_ gene was detected using a probe-based qPCR assay with the *bla*_AIM-1_ primer set and probe sequence HEX_CCATATCCTGGTCGATGCCGCC_BHQ-1 using the Taqman Fast Advanced Master Mix (Life Technologies, Foster City, CA, United states). A negative control containing all the components of the PCR mixture except for the DNA template and a positive control of genomic DNA of PA0545 (verified to be carrying *bla*_AIM-1_ by WGS) was included in every run. Results of the amplification were interpreted as positive if the amplification was between 10–30 cycles for the gene of interest and above 31 cycles for the *rspL* internal control gene [38]. Verification of the probe-based qPCR product was confirmed by Sanger sequencing (Australian Genomic Research Facility, Adelaide, Australia).

### Metagenomic sequencing of wastewater samples

To explore the full sequences of the gene identified using the probe-based qPCR assay, shotgun sequencing was performed via the 150 bp paired-ended Illumina sequencing platform at Macrogen (Seoul, Republic of Korea). Microbial and AMR gene composition was predicted using the SqueezeMeta pipeline [39]. Before assembly and annotation, adaptor sequences and low-quality reads of metagenome sequence reads were removed using Trimmomatic [27] and then assembled using Megahit [40]. Short contigs were removed using Prinseq [41] and protein coding sequences (CDSs) in contigs were identified using Prodigal [42]. The predicted CDSs were annotated and classified utilizing Diamond software [43] in conjunction with the EGG [44], KEGG [45] and PFAM [46] databases. AMR determinants were identified using Resfinder [33], CARD [34] and the ARG-ANNOT databases [34].

### Alignment and phylogenetic analysis

Basic local alignment search tool (blast) analysis was performed using the published nucleotide (accession: AM998375.1) and protein sequence (NG_048689.1) of AIM-1 as a query search utilizing the nucleotide (blastn) and protein (blastp) search tools (http://www.ncbi.nlm.nih.gov/BLAST/). Nucleotide and protein sequence alignments were performed using the clustal OMEGA program v.1.2.4 [47]. Genomic alignment and annotations of the genomic environment were curated manually. AIM-1-like amino acid sequences above 78 % identity were retrieved from the National Center for Biotechnology Information (NCBI) database and inspected for metadata information about the sample source, year and country (Table S2). Protein sequences were aligned using default settings and were edited using ESPript3.0 [48]. A phylogenetic tree was constructed using the deduced amino acid sequences of AIM-1 from this study and other AIM-1-like amino acid sequences available in public repositories and visualized using iTOLv4 [49].

## Results

### Incidence of carbapenem resistance

In our previous study [23], the antimicrobial susceptibility profiles of 147 *P *. *aeruginosa* isolates collected from human samples, companion animals and healthcare wastewater were determined against 5 classes of antibiotics. Of these, 32 isolates were found to be resistant to the most frequently used carbapenems, imipenem or meropenem. Imipenem- and/or meropenem-resistant isolates were further tested against the less frequently used carbapenems, doripenem and biapenem. Both doripenem and biapenem non-wild-type (non-wild-type resistance is defined in the Methods section) isolates were broadly correlated with imipenem and meropenem resistance, with 80 % of imipenem and 100 % of meropenem non-wild-type isolates also resistant against doripenem, while 73.3 and 83.3 % of the isolates were resistant to biapenem, respectively (Table S1).

### Detection of carbapenemase genes in resistant *P. aeruginosa* isolates

As acquisition of carbapenemases is considered to be of high epidemiological concern in the growing resistance to carbapenems [14], 32 carbapenem-resistant isolates from our previous study [23] were screened for common carbapenemase genes (Table 1) using conventional end-point PCR. One *P. aeruginosa* isolate from healthcare wastewater, PA0545, was found to be positive for *bla*_AIM-1_, coding for the Adelaide imipemenase carbapenemase enzyme.

### The carbapenemase carrying *P. aeruginosa* isolates are multidrug-resistant

The MICs of a *P. aeruginosa* clinical isolate (WCH6691) and the wastewater isolate (PA0545) displayed resistance to five major classes of antibiotics: cephalosporins, aminoglycosides, fluoroquinolones, carbapenems and the β-lactamase inhibitor tazobactam, while being susceptible to colistin (Table 2). A comparison of the two *bla*_AIM-1_-carrying *P. aeruginosa* isolates revealed that isolate WCH6691 displayed an even higher level of resistance, with MIC values of >256 to >512 mg l^−1^ to meropenem, imipenem, doripenem and biapenem when compared to isolate PA0545, which exhibited MIC values of 64 mg l^−1^ to these antibiotics, except for biapenem, which showed 8 mg l^−1^ (Table 2).

**Table 2.** The phenotypic resistance profile of *P. aeruginosa* isolates WCH6691 and PA0545 as compared to the *P. aeruginosa* ATCC 27853 and PAO1 control strains

	Antimicrobial MIC values (mg l^−1^)*											
Isolates ECOFF value	FEP 8	CAZ 8	PTZ 16	CIP 0.5	LEV 3	TOB 2	MER 2	IMI 4	DOR 1	BIA 4†	COL 4	SXT nd
ATCC 27583	2	4	4	0.5	1	0.25	1	4	1	2	1	64
PAO1	2	2	4	0.125	1	0.5	2	4	1	2	2	128
WCH6691 (clinical)	32	32	>128	16	16	32	**>512**	**>512**	>256	>256	2	>512
PA0545 (wastewater)	64	256	128	32	32	512	**64**	**64**	64	8	0.5	>512

*EUCAST epidemiological cut-off (ECOFF) value (mg l^−1^).

†MIC >4 mg l^−1^ according to a previous study [25]; MIC value in bold indicates a difference in meropenem and imipenem resistance in *P. aeruginosa* isolates harbouring *bla*_AIM-1_ genes; nd, ECOFF not defined by EUCAST.

BIA, biapenem; CAZ, ceftazidime; CIP, ciprofloxacin; COL, colistin; DOR, doripenem; FEP, cefepime; IMI, imipenem; LEV, levofloxacin; MER, meropenem; PTZ, piperacillin–tazobactam; SXT, trimethoprim–sulfamethoxazole; TOB, tobramycin.

### A genomic comparison of the *P. aeruginosa* PA0545 and WCH6691 genomes reveals different sequence types

To obtain a deeper insight into the potential genomic basis for the higher level of phenotypic carbapenem resistance detected in the clinical strain WCH6691 compared to isolate PA0545, both isolates were subjected to WGS. A genomic comparison of the two isolates revealed a genome size of 6.8 and 7.1 Mbp for the WCH6691 and PA0545 isolates with a G+C content of 66.06 and 65.42 %, respectively. An analysis of the MLST revealed that they belong to different sequence types (STs), with the clinical isolate (WCH6691) belonging to an international high-risk epidemic clone, ST235, while the wastewater isolate (PA0545) belongs to an uncommon but multidrug-resistant ST815 clone (Table 3).

**Table 3.** Source, sequence type and antimicrobial resistance determinants of *P. aeruginosa* isolates WCH6691 and PA0545.

				β-lactam drug resistance gene determinants	Aminoglycoside resistance gene determinants	Fluoroquinolone resistance determinants				SXT	Efflux pump genes
Isolate	Source	Year	MLST			GyrA	ParC	ParE	CrpP		
WCH6691	Human	2016	235	AIM-1^⁎^, PDC-35†, OXA-488‡	*aadA6*, APH (3′)-IIb*, aac (3)-IIe*	T83I	S87L	D533E	−	*sul1*	
PA0545	WW	2017	815	AIM-1, SHV-5, PDC-100, OXA-50	*aadA, aadA6*, APH (3′)-IIb, *aac (3)-Ia*, *aac (3)-IIe*	D87N	V297I	D533E	*+*	*dfrA1, sul1*	*qacE delta1*

*Carbapenemases; †AmpC-β-lactamase; ‡extended-spectrum β-lactamase; WW, wastewater. SXT, trimethoprim–sulfamethoxazole.

Analysis of AMR determinants present in WCH6691 and PA0545 revealed that both isolates harbour identical *bla*_AIM-1_ genes (Fig. S1, available in the online version of the article), although differences in other resistance determinants were detected. In addition to the *bla*_AIM-1_ gene, the two isolates carried the chromosomal β-lactamase PDC variants, *bla*_OXA-50_ variants, the aminoglycoside-modifying genes *aadA6*, APH (*3′)-IIb* and *aac (3)-IIe* and the sulfonamide resistance gene *sul1* (Table 3). Resistance determinants unique to PA0545 included: *bla*_SHV-5_, an extended-spectrum cephalosporinase, the aminoglycoside-modifying enzymes *aadA* and *aac (3)-Ia*, the trimethoprim resistance gene *dfrA1*, the plasmid-mediated ciprofloxacin resistance gene *crpP* and the efflux pump gene *qacEdelta1*. Amino acid mutations were also observed in the quinolone resistance-determining regions of the GyrA protein T83I and ParC S87L in WCH6691 and D87N and V297I in PA0545 (Table 3).

**Fig. 1.**
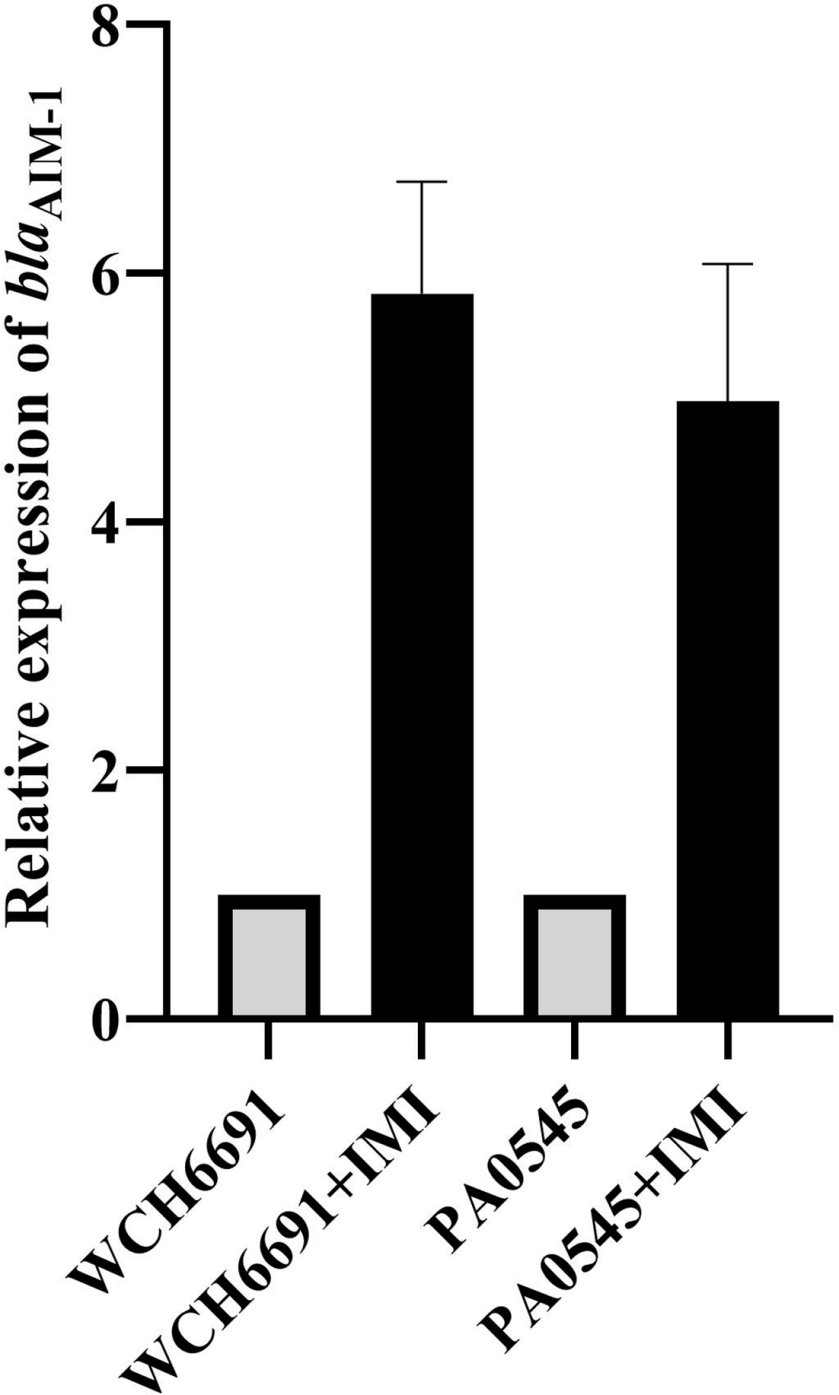
The expression levels of the *bla*_AIM-1_ genes are similar in the WCH6691 and PA0545 *P. aeruginosa* isolates. The relative expression of the *bla*_AIM-1_ genes after imipenem shock (black bars) are indicated as a fold increase compared to the baseline expression level in the absence of imipenem (grey bars). The baseline expression of *bla*_AIM-1_ was the same in both isolates (2.65±0.2) and was taken as 1. The error bars show the standard deviation of three technical replicates of each.

Since no differences were detected between the *bla*_AIM-1_ nucleotide sequences of the two isolates, the reason for the difference in the phenotypic carbapenem resistance was further investigated. The possibility of increased expression levels of the *bla*_AIM-1_ gene in the clinical WCH6691 isolate were considered. To test this hypothesis, the relative expression level of *bla*_AIM-1_ in the two isolates was assessed. The expression levels of *bla*_AIM-1_ in the absence of any carbapenem stress were similar/identical. The expression level of *bla*_AIM-1_ increased by almost sixfold in both isolates in the presence of the carbapenem antibiotic imipenem (Fig. 1).

### Analysis of additional carbapenem resistance mechanisms

**Fig. 2.**
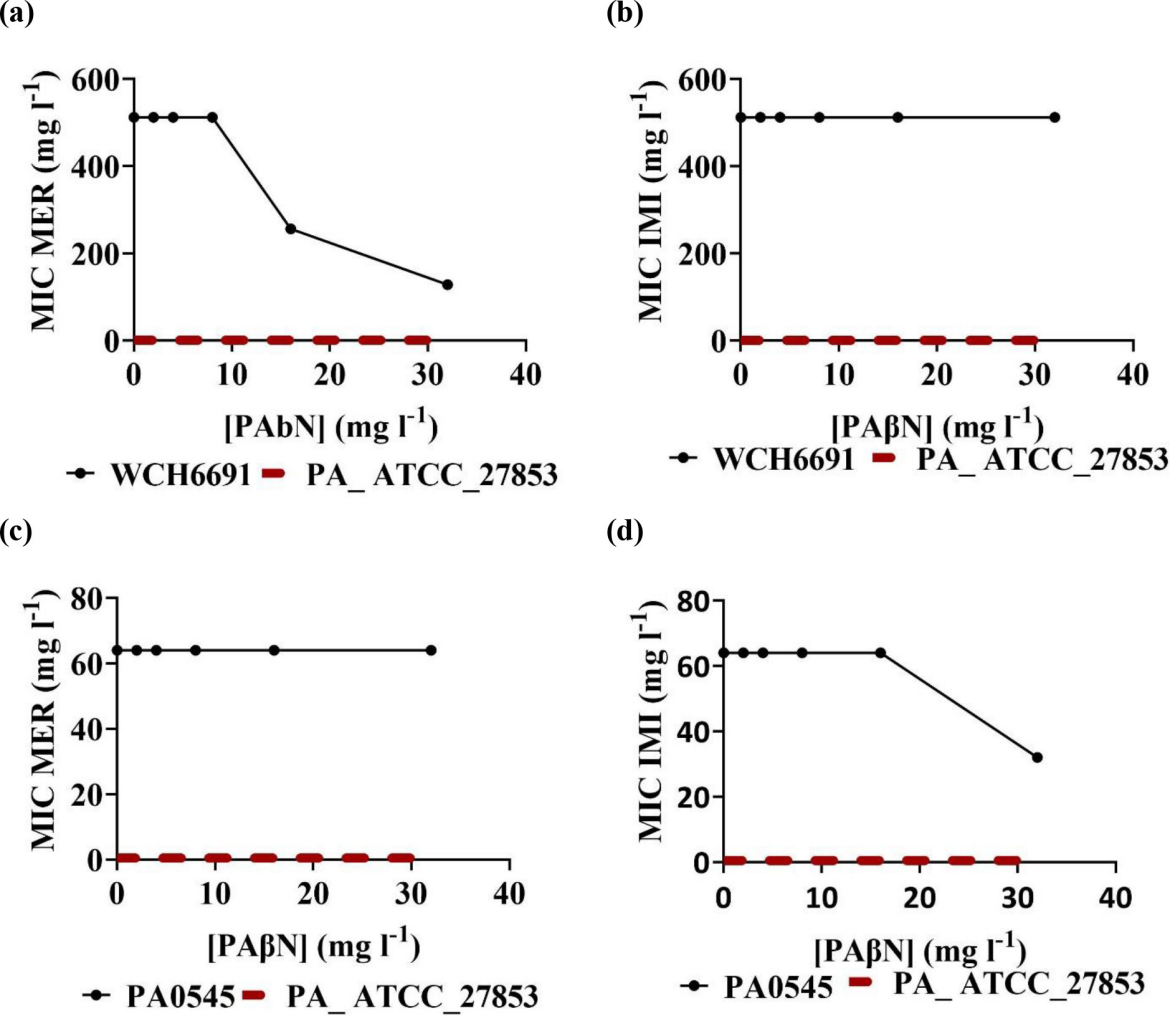
Efflux pumps contribute to high-level meropenem but not imipenem resistance. Data represent the MIC of *P. aeruginosa* ATCC27853 control strain (red dashed line) at MIC=0.5 mg l^−1^ and the MIC of each isolate tested in the presence of PAβN (black line). MIC, minimum inhibitory concentration; MER, meropenem; IMI, imipenem; PAβN, phenylalanine arginine β-naphthylamide.

Since the nucleotide sequences of the *bla*_AIM-1_ genes were identical and there was no difference in the gene expression level (*P*=0.352) between the *P. aeruginosa* WCH6691 and PA0545 isolates, other factors must be responsible for the higher phenotypic carbapenem resistance levels observed in isolate WCH6691 when compared to isolate PA0545. Resistance to carbapenem is often mediated through a combination of resistance mechanisms, including mutations that decrease antibiotic uptake and overexpression of both efflux pumps and antibiotic inactivation enzymes [12]. To investigate the role of RND efflux pumps in conferring imipenem and meropenem resistance, the MICs of these two antibiotics were determined in the presence of the RND efflux pump inhibitor, PAβN (Fig. 2a–d). A significant (≥4-fold) reduction in the MIC of meropenem was observed for isolate WCH6691 in the presence of the efflux pump inhibitor. However, no significant reduction in MIC in the presence of the efflux pump inhibitor was observed for imipenem (Fig. 2a–d). Hence, antibiotic efflux could explain the higher level of resistance observed of isolate WCH6691 for meropenem but not for imipenem.

To further explore the difference in imipenem resistance levels observed between the two isolates, bioinformatic analysis with a focus on antibiotic uptake was undertaken. A comparison of the OprD protein sequences of WCH6691 and PA0545 isolates to the *P. aeruginosa* PAO1 reference strain (accession number: NP_249649.1) (Fig. S2) revealed several sequence variations in the OprD protein (Table S2). The majority of these sequence variants are located in the external loops of the OprD protein, which have been shown to be responsible for the binding of carbapenems; however, most of these have been shown not to be associated with carbapenem resistance [50]. Nevertheless, sequence variants such as F170L, found in OprD in the WCH6691 isolate, have previously been described and have been associated with carbapenem resistance [51], hence this variation could provide a possible explanation for the increased phenotypic resistance observed for imipenem in this isolate.

### Expanding the search of the carbapenem resistance *bla*_AIM-1_ gene in Adelaide

The increased activity of the AIM-1 enzyme and the high level of resistance it confers to carbapenem antibiotics, could seriously affect the ability to reverse resistance, which is conferred by this enzyme via the use of carbapenem–carbapenemase inhibitor drug combinations. This resistance determinant is not included in surveillance programmes due to its perceived restricted geographical occurrence. It is therefore difficult to ascertain the incidence and spread of the *bla*_AIM-1_ gene [52]. As we detected this gene in an isolate from healthcare wastewater origin, we extended our search to assess the environmental occurrence of the *bla*_AIM-1_ gene in wastewater from other healthcare facilities (*n*=6 sites), from the community (*n*=3 sites) and in water from a local river (*n*=1 site), utilizing probe-based qPCR. All the wastewater samples, as well as the river sample were positive for the *bla*_AIM-1_ gene. The identity of all PCR-positive products was confirmed to be the *bla*_AIM-1_ gene with Sanger sequencing.

### Nucleotide and protein database searching revealed the worldwide distribution of the *bla*_AIM-1_ gene

The occurrence of *bla*_AIM-1_ throughout Adelaide, as observed in the wastewater and river samples tested, hinted at a more widespread distribution than previously assumed. Therefore, the possibility of a global distribution of the *bla*_AIM-1_ gene was investigated. A blast search using both the nucleotide (accession number: WP_063857820.1) and the amino acid sequence of AIM-1 derived from WP_063857820.1 as a query was performed. The results revealed that the AIM-1 determinant was detected in the Middle East (Iraq), Asia, North America and Europe. Further investigation of the source/carriage of the *bla*_AIM-1_ gene was noted predominantly in the ubiquitous environmental bacterium, *P. mexicana* (Table S3) and in *P. aeruginosa* in Iraq, which was not previously described [53].

**Fig. 3.**
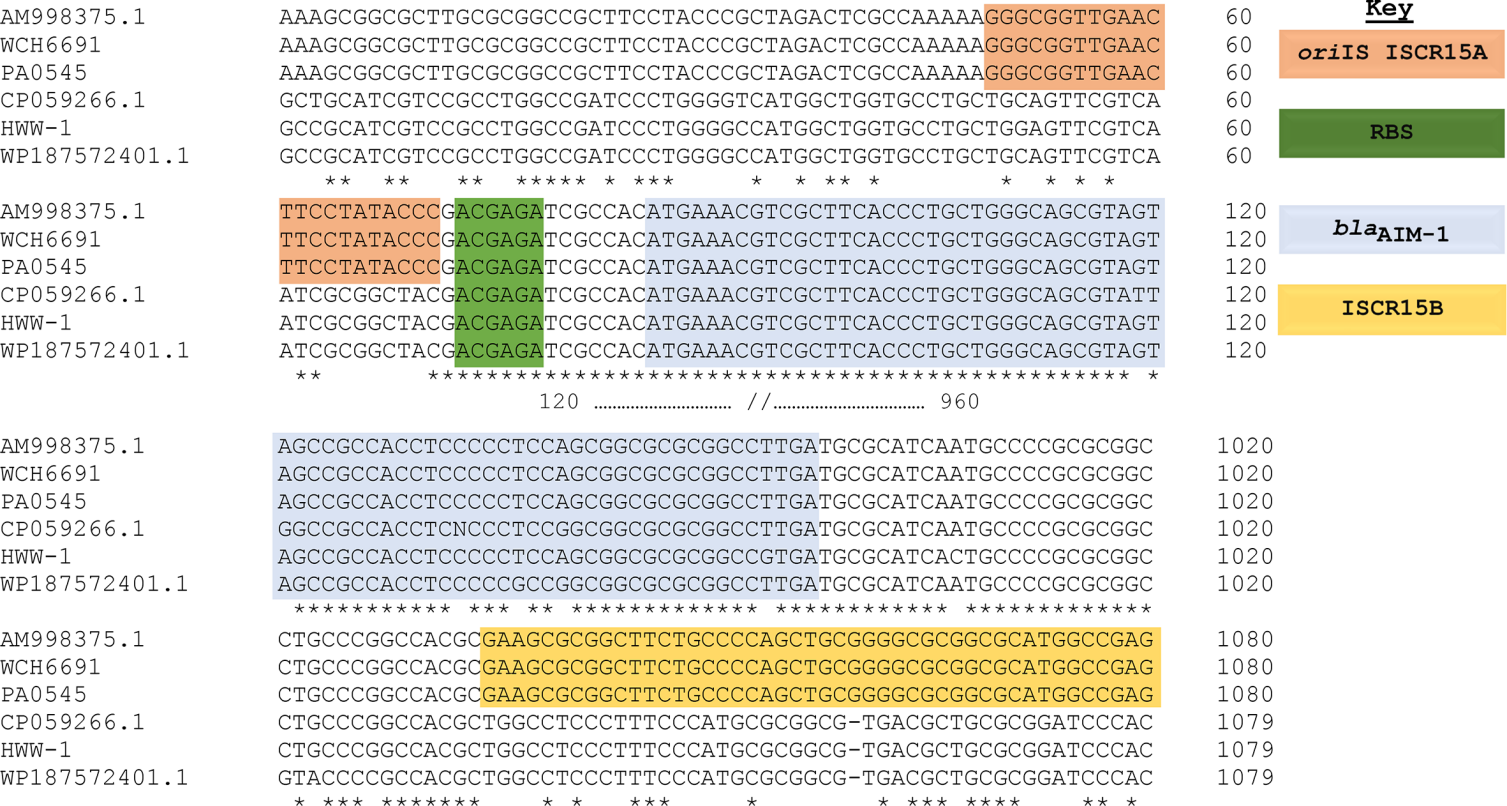
The *bla*_AIM-1_ genomic environment of the HWW-1 wastewater sample is similar to that of *P. mexicana *, lacking the ISCR15 mobile genetic element that is present in *P. aeruginosa *. Nucleotide sequence alignment of closely related *bla*_AIM-1_ genes from *P. aeruginosa* PA0545, WCH6691, the HWW-1 wastewater sample, the first identified *P. aeruginosa* carrying the *bla*_AIM-1_ gene in Adelaide, Australia (accession number AM998375.1) and from *P. mexicana* isolated in Singapore (accession number CP059266.1) and PR China (accession number WP187572401.1). The genetic environments of each isolate as determined by a multiple sequence alignment revealed an origin of insertion sequence (oriIS) of ISCR15A (highlighted in orange) and ISCR15B (golden) in *P. aeruginosa *, which are shown to be absent in the HWW-1 wastewater sample and in *P. mexicana *. All sequences have a similar ribosomal-binding site (RBS) (green) and *bla*_AIM-1_ gene sequence (blue). The downstream region of the *bla*_AIM-1_ gene also shows a near perfect sequence identity between these sequences (37 nucleotide sequences between the 3′ end of the *bla*_AIM-1_ gene and ISCR15B). Dashes represent nucleotides that are lacking in the indicated sequence and asterisks indicate identical nucleotides.

Although the primer/probe-based qPCR could verify the presence of the gene in all tested wastewater and river water assayed in this study, the identity of the organism(s) carrying this gene could not be determined. To elucidate the identity of the organism(s) carrying the gene and to assess the genomic arrangement of the *bla*_AIM-1_ gene, shotgun metagenomic sequencing was conducted on wastewater sourced from two healthcare settings (HWW-1 and 2) and one non-healthcare (NHWW-1) setting. All three sites returned positive for the *bla*_AIM-1_ gene, with the sequence from HWW-1 returning a clean contig containing the *bla*_AIM-1_ gene and surrounding features. This contig was 2478 bp long and the entirety was identified as originating from *P. mexicana *. Comparison of this contig with that from *P. aeruginosa* isolates WCH6691, PA0545 and AM998375.1 (nucleotide sequence deposited into the NCBI showing the presence of an ISCR15) sequences revealed that, unlike the genetic environment and arrangement in *P. aeruginosa *, the *bla*_AIM-1_ gene was not carried as part of a transposon, or any other mobile genetic element in the HWW-1 wastewater sample (Fig. S1). For comparison, the *bla*_AIM-1_ genes from *P. mexicana* were also investigated. Similarly to the wastewater sample from HWW-1, no mobile genetic elements were detected upstream or downstream of the *bla*_AIM-1_ gene in the *P. mexicana* isolates (Fig. 3).

**Fig. 4.**
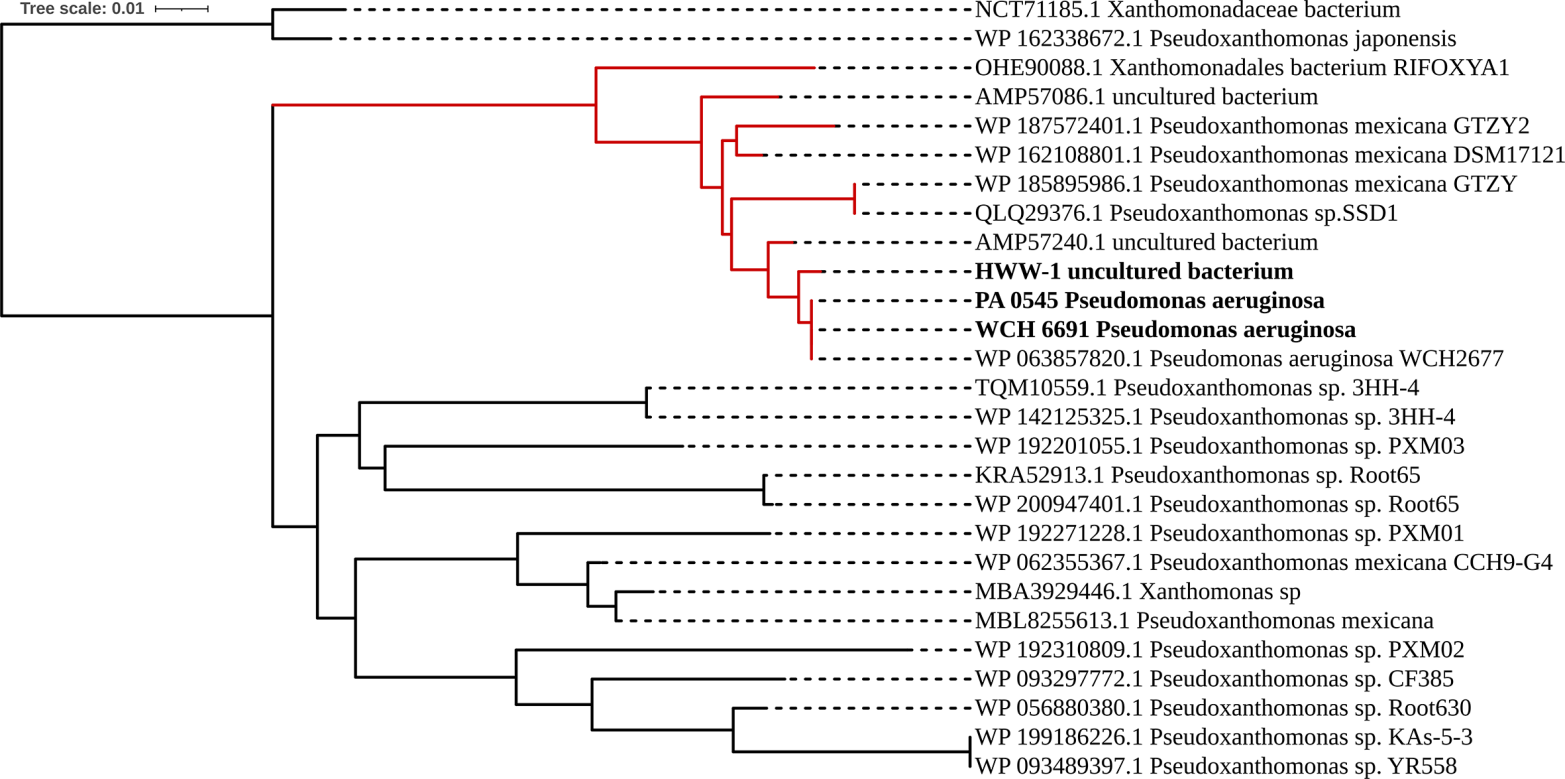
Phylogenetic analysis revealed that the AIM-1 identified in this study clustered with AIM-1 amino acid sequences from *P. mexicana *. The AIM-1 amino acid sequences assessed in this study (boldface) are clustered together with the initially identified AIM-1 amino acid sequence from *P. aeruginosa* (accession number WP063857820.1) and *P. mexicana* retrieved from the NCBI database (red branches); the geographical location, source and year of isolation for AIM-1 proteins from *P. mexicana* retrieved from NCBI are provided in Table S3. The analysis was performed using the neighbour-joining tree model and the tree was annotated using iTOLv4 [49].

To evaluate the relatedness of the AIM-1 variants sequenced in this study with the variant initially reported in *P. aeruginosa* (accession number: WP_063857820.1) [21] and with the variants carried by *Pseudoxanthomonas* species genomes available in the public databases (Table S3), a phylogenetic tree was constructed based on the deduced amino acid sequences of AIM-1. The phylogenetic analysis revealed three clusters (Fig. 4). The AIM-1 variants identified in this study were clustered together with the AIM-1 proteins from the *P. aeruginosa* initially identified in Adelaide, Australia and an environmental bacterium, *P. mexicana *.

### *P. mexicana* is the possible origin of the *bla*_AIM-1_ gene

In this study, and in the initial report of *bla*_AIM-1_ in *P. aeruginosa* [21], the genetic environment of the *bla*_AIM-1_ gene carried by *P. aeruginosa* isolates revealed the presence of the ISCR15, which was absent in *P. mexicana *. However, assessment of the genetic environments carried out by a multiple sequence alignment on the phylogenetically related *bla*_AIM-1_ genes revealed a similar *ori*IS in *P. aeruginosa* and the RBS upstream of the *bla*_AIM-1_ gene start codon in both *P. aeruginosa* and *P. mexicana *. Evaluation of the downstream region also revealed a near-perfect sequence identity between sequences from these organisms (Fig. 3). Finally, the active site of the protein appears to be well conserved between isolates. However, it is difficult to speculate what the native function of the AIM-1 protein is in *P. mexicana* without further study (Fig. S3). Altogether, these results strongly suggested that the potential origin of the *bla*_AIM-1_ gene may be the non-pathogenic ubiquitous environmental organism, *P. mexicana *.

## Discussion

The combination of phenotypic and genotypic-based approaches used here to characterize MDR *P. aeruginosa* carrying the *bla*_AIM-1_ gene has provided an interesting outlook on the clinical relevance, global widespread occurrence, and origin/source of this gene. The phenotypic profile of the two *P. aeruginosa* isolates (WCH6691 and PA0545) carrying *bla*_AIM-1_ displayed MDR, including resistance to cephalosporins, fluoroquinolones, aminoglycosides, carbapenems and piperacillin–tazobactam (β-lactamase inhibitor). In contrast, both isolates remain wild-type to the last-resort antibiotic colistin. WGS further elucidated the resistance determinants responsible for this MDR classification. Although both isolates revealed a similar level of *bla*_AIM-1_ overexpression, the clinical isolate WCH6691 displayed high-level resistance to carbapenems compared to the wastewater isolate. This difference could be explained by the presence of additional resistance mechanisms, such as efflux pumps for meropenem and doripenem, and resistance and mutations (F170L) associated with carbapenem resistance in OprD [54] in the clinical isolate. A comparison of the resistance profiles of both isolates with a focus on other cephalosporins particularly ceftazidime revealed that the wastewater isolate PA0545 displayed a higher level of resistance than the clinical isolate. This could be attributed to the presence of an additional cephalosporin-resistant extended-spectrum β-lactamase (ESBL), the SHV-5 protein, which is more common in *Enterobacterales* than in *P. aeruginosa* [55].

The isolates belonged to two different sequence types (STs), with WCH6691 belonging to an international high-risk epidemic ST, ST235, and PA0545 belonging to an uncommon but multidrug-resistant ST, ST815. ST235 represents the third most frequent ST in the *P. aeruginosa* population and is mostly isolated from hospitalized patients and is associated with poor clinical outcomes, partly due to their multi- and high-level AMR [56]. In contrast, the wastewater isolate, PA0545, belongs to an uncommon ST, ST815, which carries more mobile antimicrobial resistance genes than the clinical isolate WCH6691. Multidrug-resistant *P. aeruginosa* carrying a carbapenemase and other mobile antibiotic resistance genes, such as *bla*_SHV-5_, *aac(3)-Ia, aac (3)-IIe*, *crpP*, *dfrA1* and *qacEdelta1*, was isolated from a hot spot healthcare wastewater environment. This could be an indication of the selective advantage for the emergence of this high-level MDR uncommon ST in healthcare-associated wastewater [57].

There is now increasing evidence showing that clinically relevant novel carbapenemases originate from environmental bacteria [58]. The exchange of genetic material between these and clinically important bacteria is not surprising, as they share the same environmental niches [59]. The transmission and dissemination of carbapenem resistance genes are accelerated when the genes are carried by mobile genetic elements such as plasmids and insertion sequence elements [60]. The *bla*_AIM-1_ genes detected in our clinical and wastewater *P. aeruginosa* isolates were flanked by the insertion sequence, ISCR15, which matches the originally identified genetic environment. However, the *bla*_AIM-1_ gene detected in the healthcare wastewater sample (HWW-1) demonstrated a lack of the ISCR15 element and corresponded to the *P. mexicana* sequence. Analysis also revealed that the RBSs in the upstream region of the *bla*_AIM-1_ genes were identical in all assessed sequences. Additionally, an almost identical nucleotide sequence in the downstream region of the *bla*_AIM-1_ gene in both *P. aeruginosa* and *P. mexicana* was observed. Finally, amino acid analysis revealed that the residues involved in the active binding sites were conserved amongst all isolates and were phylogenetically clustered. Combined, these results strongly suggested that the potential origin of the *bla*_AIM-1_ gene may be the non-pathogenic ubiquitous environmental organism, *P. mexicana *.

All five of the currently available *P. mexicana* genomes in the NCBI carry the *bla*_AIM-1_ gene. Of these, three sequences phylogenetically clustered with our AIM-1 protein. This suggested that AIM-1 is intrinsic in the widespread harmless organism, *P. mexicana,* which shares similar environmental niches with *P. aeruginosa. P. mexicana* is an environmental free-living bacterium present in soil, ground water, wastewater and even in anaerobic digestor treatment plants [61]. So far, there is no evidence that this bacterium is pathogenic to humans [59]. However, since carbapenems are naturally occurring antibiotics produced by the soil organism *Streptomyces cattleya* [62], it is tempting to speculate that environmental *P. mexicana,* which is capable of producing an enzyme to degrade this antibiotic, would have a selective advantage. Furthermore, it is likely that the selective pressure in a wastewater environment could further increase the mobilization of the *bla*_AIM-1_ gene into other potential clinically important bacterial isolates both within the healthcare settings and in the surrounding environment. Despite the high-level resistance phenotype associated with this gene and its widespread presence in environmental, non-pathogenic bacteria, it rarely becomes established in pathogenic bacterial populations. *P. aeruginosa* carrying the *bla*_AIM-1_ gene has been infrequently, though repeatedly, reported in a specific region (Adelaide, South Australia); however, recent clinical *P. aeruginosa* isolates carrying this gene have also been reported in Iran [11] and Iraq [63] implying that the mobilization of the *bla*_AIM-1_ gene is not simply a localized phenomenon.

Our study clearly shows that the presence of AIM-1 affords *P. aeruginosa* high carbapenem resistance and the spread of *bla*_AIM-1_ is currently occurring at the same time in various dimensions, since it can now be found in different geographical locations, in other environmental bacterial species and in different environmental settings. An increased understanding of the selective pressures that encourage these transfer events would enable mitigation of the risks of the *bla*_AIM-1_ gene transfer to pathogenic species.

## Supplementary Data

Supplementary material 1
