# Supplementary material for: Worldwide distribution and environmental origin of the Adelaide imipenemase (AIM-1), a potent carbapenemase in Pseudomonas aeruginosa
Source: Microb Genom. 2021 Dec 17;7(12):000715. doi: 10.1099/mgen.0.000715 (PMC8767344; doi:10.1099/mgen.0.000715)
Supplement: Supplementary material 1 [file mgen-7-0715-s001.pdf]

Worldwide distribution and environmental origin of a potent carbapenemase, the Adelaide Imipenemase (AIM-1)

Table S1. Percentage of carbapenem resistance isolates and effect of an efflux pump inhibitor on MIC of carbapenem resistance *P. aeruginosa* isolates

	MIC [mg/L) in the presence and absence of PaβN							
Isolates ID	**MER**	**MER+PAβN**	**Fold reduction**	**IMP**	**IMI+PAβN**	**Fold reduction**	**Doripem**	**Bipenem**
PA0382	4	2	2	32	16	2	4	8
PA0404	2	ND	ND	16	8	2	1	4
PA0461	4	ND	ND	32	16	2	8	8
PA0468	4	ND	ND	8	8	1	8	8
PA0471	4	ND	ND	8	8	1	2	8
PA0532	8	1	8	16	8	2	4	8
PA0533	4	ND	ND	64	64	1	2	2
PA0536	64	64	1	64	64	1	>32	4
PA0545	64	64	1	64	32	2	>256	8
PA0546	8	4	2	8	8	1	2	4
CLN02	2	ND	ND	8	8	1	2	8
CLN06	4	ND	ND	2	ND	ND	ND	ND
CLN07	0.5	ND	ND	8	8	1	0.5	1
CLN13	4	0.5	8	64	64	1	4	16
CLN14	32	16	2	32	32	1	8	16

CLN22	0.5	ND	ND	16	8	2	1	1
CLN28	32	32	1	64	64	1	32	32
CLN29	8	8	1	32	8	4	4	8
CLN30	8	1	8	32	8	4	8	16
CLN32	32	8	4	32	8	4	32	32
CLN33	8	8	1	32	32	1	8	16
CLN41	16	16	1	32	8	4	16	16
CLN44	1	ND	ND	8	8	1	1	8
CLN58	4	ND	ND	4	ND	ND	ND	ND
CLN65	2	ND	ND	16	8	2	2	16
CLN66	16	8	2	16	8	2	8	8
CLN71	16	8	2	8	8	1	8	8
CLN77	16	2	8	32	8	4	8	32
CLN81	4	ND	ND	8	8	1	2	16
CLN84	2	ND	ND	8	8	1	0.125	0.5
CLN86	2	ND	ND	16	8	2	1	8
CLN88	4	4	1	8	8	1	4	1
**Total resistance**	**24/147 (16.3%)**			**30/147 (20.4%)**			**24/30 (80%)**	**22/30 (73.3%)**

Table S2. Comparison of the OprD and PDC amino acid mutations of WCH6691and PA0545 *P. aeruginosa* isolates to that of the wild-type OprD and PDC-1 variant from *P. aeruginosa* PAO1.

Isolates	OprD mutations	PDC mutations
**WCH6691 clinical**	81 amino acid truncation (M1 to T81), V82M, T103S, K115T, F170L*, **E185Q, P186G, V189T, R310E, A315G**, G425A	G27D, **V97A, T105A*, V205L, G391A**
**PA0545 wastewater**	V127L, M135I**, E185Q, P186G, V189T**, E202Q, I210A, E230K, S240T, N262T, T276A, A281G, K296Q, Q301E**, R310E, A315G**, L347M, S403A, Q424E, 372V-DSSSS..YAGL-383 (loop 7 short)**	T21A, **V97A, T105A***, Q155R, A156V, **V205L**, S306T**, G391A**

*mutations have previously been described in carbapenem resistance in *P. aeruginosa* and have been associated with carbapenem-resistance (51). **shortening of loop 7 has been previously reported (54). Variations indicated in bold are considered common amino acid substitutions. PDC, *Pseudomonas*-derived cephalosporinase

```
AM998375.1      AAAGCGGCGCTTGCGCGGCCGCTTCCTACCCGCTAGACTCGCCAAAAAGGGCGGTTGAAC   60
WCH6691         AAAGCGGCGCTTGCGCGGCCGCTTCCTACCCGCTAGACTCGCCAAAAAGGGCGGTTGAAC   60
PA0545          AAAGCGGCGCTTGCGCGGCCGCTTCCTACCCGCTAGACTCGCCAAAAAGGGCGGTTGAAC   60
CP059266.1      GCTGCATCGTCCGCCTGGCCGATCCCTGGGGTCATGGCTGGTGCCTGCTGCAGTTCGTCA   60
HWW-1           GCCGCATCGTCCGCCTGGCCGATCCCTGGGGCCATGGCTGGTGCCTGCTGGAGTTCGTCA   60
WP187572401.1   GCCGCATCGTCCGCCTGGCCGATCCCTGGGGCCATGGCTGGTGCCTGCTGCAGTTCGTCA   60
                   **  **   **  ***** * ***     *  * ** *        *  * * *

AM998375.1      TTCCTATACCCGACGAGATCGCCACATGAAACGTCGCTTCACCCTGCTGGGCAGCGTAGT   120
WCH6691         TTCCTATACCCGACGAGATCGCCACATGAAACGTCGCTTCACCCTGCTGGGCAGCGTAGT   120
PA0545          TTCCTATACCCGACGAGATCGCCACATGAAACGTCGCTTCACCCTGCTGGGCAGCGTAGT   120
CP059266.1      ATCGCGGCTACGACGAGATCGCCACATGAAACGTCGCTTCACCCTGCTGGGCAGCGTATT   120
HWW-1           ATCGCGGCTACGACGAGATCGCCACATGAAACGTCGCTTCACCCTGCTGGGCAGCGTAGT   120
WP187572401.1   ATCGCGGCTACGACGAGATCGCCACATGAAACGTCGCTTCACCCTGCTGGGCAGCGTAGT   120
                 **       ************************************************ *

AM998375.1      CGCCCTCGCCCTCTCATCCACAGCCCTCGCCTCCGATGCGCCCGCCTCCAGGGGCTGCGC   180
WCH6691         CGCCCTCGCCCTCTCATCCACAGCCCTCGCCTCCGATGCGCCCGCCTCCAGGGGCTGCGC   180
PA0545          CGCCCTCGCCCTCTCATCCACAGCCCTCGCCTCCGATGCGCCCGCCTCCAGGGGCTGCGC   180
CP059266.1      CGCCCTCGCCCTTTCATCCGCCACCCTCGCCTCCGATGCGCCCGCCTCCAGGGGCTGCGC   180
HWW-1           CGCCCTCGCCCTCTCATCCACAGCCCTCGCCTCCGATGCGCCCGCCTCCAGGGGCTGCGC   180
WP187572401.1   CGCCCTCGCCCTCTCATCCACAGCCCTCGCCTCCGATGCGCCTGCCTCCAGGGGCTGCGC   180
                ************ ****** *  ******************* *****************

AM998375.1      CGACGATGCCGGCTGGAACGATCCGGCAATGCCCCTGAAGGTGTACGGAAACACCTGGTA   240
WCH6691         CGACGATGCCGGCTGGAACGATCCGGCAATGCCCCTGAAGGTGTACGGAAACACCTGGTA   240
PA0545          CGACGATGCCGGCTGGAACGATCCGGCAATGCCCCTGAAGGTGTACGGAAACACCTGGTA   240
CP059266.1      CGACGATGCGGGCTGGAACGATCCGGCAACGCCCCTGAAGGTGTACGGCAACACCTGGTA   240
HWW-1           CGACGATGCCGGCTGGAACGATCCGGCAATGCCCCTGAAGGTGTACGGAAACACCTGGTA   240
WP187572401.1   CGACGATGCGGGCTGGAACGATCCGGCAACGCCCCTGAAGGTGTATGGCAACACCTGGTA   240
                ********* ******************* *************** ** ***********

AM998375.1      CGTTGGCACCTGCGGCATCAGTGCGCTGCTGGTCACTTCCGACGCGGGCCATATCCTGGT   300
WCH6691         CGTTGGCACCTGCGGCATCAGTGCGCTGCTGGTCACTTCCGACGCGGGCCATATCCTGGT   300
PA0545          CGTTGGCACCTGCGGCATCAGTGCGCTGCTGGTCACTTCCGACGCGGGCCATATCCTGGT   300
CP059266.1      TGTCGGCACCTGCGGCATCAGTGCGCTGCTGGTCACGTCCGACGCGGGCCATATCCTGGT   300
HWW-1           CGTTGGCACCTGCGGCATCAGTGCGCTGCTGGTCACTTCCGACGCGGGCCATATCCTGGT   300
WP187572401.1   CGTCGGCACCTGCGGCATCAGTGCGCTGCTGGTCACTTCCGACGCGGGCCATATCCTGGT   300
                 ** ******************************** ***********************
```

Key

- ***ori*IS ISCR15A**
- **RBS**
- ***bla*AIM-1**
- **ISCR15B**

```
AM998375.1      CGATGCCGCCACGCCGCAGGCGGGCCCACAGATCCTGGCCAACATCCGCGCACTCGGTTT   360
WCH6691         CGATGCCGCCACGCCGCAGGCGGGCCCACAGATCCTGGCCAACATCCGCGCACTCGGTTT   360
PA0545          CGATGCCGCCACGCCGCAGGCGGGCCCACAGATCCTGGCCAACATCCGCGCACTCGGTTT   360
CP059266.1      CGATGCCGCCACGCCGCAGGCGGGCCCGCAGATCCTGGCCAACATCCGCGCGCTCGGCTT   360
HWW-1           CGATGCCGCCACGCCGCAGGCGGGCCCGCAGATCCTGGCCAACATCCGCGCACTCGGTTT   360
WP187572401.1   CGATGCCGCCACGCCGCAGGCGGGCCCGCAGATCCTGGCCAACATCCGCGCACTCGGTTT   360
                *************************** *********************** ***** **

AM998375.1      CAGGCCGGAGGACGTGCGCGCCATCGTGTTCTCGCACGAGCATTTCGACCATGCCGGCAG   420
WCH6691         CAGGCCGGAGGACGTGCGCGCCATCGTGTTCTCGCACGAGCATTTCGACCATGCCGGCAG   420
PA0545          CAGGCCGGAGGACGTGCGCGCCATCGTGTTCTCGCACGAGCATTTCGACCATGCCGGCAG   420
CP059266.1      CAAGCCGGAGGACGTGCGCGCCATCGTGTTCTCGCACGAGCATTTCGACCATGCCGGCAG   420
HWW-1           CAGGCCGGAGGACGTACGGGCCATCGTGTTCTCGCACGAGCATTTCGACCATGCCGGCAG   420
WP187572401.1   CAGGCCGGAGGACGTACGGGCCATCGTGTTCTCGCACGAGCATTTCGACCATGCCGGCAG   420
                ** ************ ** *****************************************

AM998375.1      CCTCGCCGAACTGCAGAAGGCCACGGGTGCACCGGTGTACGCGCGCGCGCCCGCGATCGA   480
WCH6691         CCTCGCCGAACTGCAGAAGGCCACGGGTGCACCGGTGTACGCGCGCGCGCCCGCGATCGA   480
PA0545          CCTCGCCGAACTGCAGAAGGCCACGGGTGCACCGGTGTACGCGCGCGCGCCCGCGATCGA   480
CP059266.1      CCTCGCCGAACTGCAGAAAGCCACGGGTGCACCGGTGTACGCGCGCGCNCCNGCGATCGA   480
HWW-1           CCTCGCAGAACTGCAGAAGGCCACGGGCGCACCGGTGTATGCGCGCGCGCCCGCGGTCGA   480
WP187572401.1   CCTCGCCGAACTGCAGAAGGCCACGGGCGCACCGGTGTATGCGCGCGCGCCCGCGGTCGA   480
                ****** *********** ******** *********** ******** ** *** ****

AM998375.1      CACGCTGAAGCGCGGCCTGCCGGACCGCACCGACCCGCAATTCGAGGTGGCCGAACCCGT   540
WCH6691         CACGCTGAAGCGCGGCCTGCCGGACCGCACCGACCCGCAATTCGAGGTGGCCGAACCCGT   540
PA0545          CACGCTGAAGCGCGGCCTGCCGGACCGCACCGACCCGCAATTCGAGGTGGCCGAACCCGT   540
CP059266.1      CACGCTGAAGCGCGGCCTGCCGGACCGCACCGACCCGCAGTTCGAGGTGGCCGAACCCAT   540
HWW-1           CACGTTGAAGCGCGGCCTGCCGGACCGCACCGACCCGCAATTCGAGGTGGCCGAACCCGT   540
WP187572401.1   CACGTTGAAGCGCGGCCTGCCGGACCGCACCGACCCGCAGTTCGAGGTGGCCGAACCCAT   540
                **** ********************************** ****************** *

AM998375.1      TGCGCCGGTCGCCAACATCGTCACCCTGGCCGACGACGGCGTGGTGAGCGTCGGCCCGCT   600
WCH6691         TGCGCCGGTCGCCAACATCGTCACCCTGGCCGACGACGGCGTGGTGAGCGTCGGCCCGCT   600
PA0545          TGCGCCGGTCGCCAACATCGTCACCCTGGCCGACGACGGCGTGGTGAGCGTCGGCCCGCT   600
CP059266.1      CGCGCCGGTCGCCAACATCGTCGCCCTGGCCGACGACGGCGTGGTGAGCGTCGGCCCGCT   600
HWW-1           CGCGCCGGTCGCCAACATCGTCACCCTGGCCGACGACGGCGTGGTGAGCGTCGGCCCGCT   600
WP187572401.1   CGCGCCGGTCGCCAACATCGTCGCCCTGGCCGACGACGGCGTGGTGAGCGTCGGTCCGCT   600
                 ********************* ******************************* *****
```

```
AM998375.1        GGCCCTGACGGCGGTCGCCTCGCCTGGCCACACCCCGGGTGGCACCAGTTGGACCTGGCG    660
WCH6691           GGCCCTGACGGCGGTCGCCTCGCCTGGCCACACCCCGGGTGGCACCAGTTGGACCTGGCG    660
PA0545            GGCCCTGACGGCGGTCGCCTCGCCTGGCCACACCCCGGGTGGCACCAGTTGGACCTGGCG    660
CP059266.1        GGCCCTGACAGCGATCGCCTCGCCTGGCCACACCCCGGGTGGCACCAGTTGGACCTGGCG    660
HWW-1             GGCCCTGACGGCGGTCGCCTCGCCTGGCCCCACCCCGGGTGGCACCAGTTGGACCTGGCG    660
WP187572401.1     GGCCCTGACGGCGGTCGCCTCGCCTGGCCACACCCCGGGAGGCACCAGTTGGACCTGGCG    660
                  ********* *** *************** ********* ********************

AM998375.1        CTCCTGCGAAGGCGACGACTGTCGCCAGATGGTCTACGCCGACAGCCTGACGGCGATCTC    720
WCH6691           CTCCTGCGAAGGCGACGACTGTCGCCAGATGGTCTACGCCGACAGCCTGACGGCGATCTC    720
PA0545            CTCCTGCGAAGGCGACGACTGTCGCCAGATGGTCTACGCCGACAGCCTGACGGCGATCTC    720
CP059266.1        CTCCTGCGAAGGCGACGACTGTCGCCAGATGGTCTACGCCGACAGCCTGACGGCGATCTC    720
HWW-1             CTCCTGCGAAGGCGACGACTGTCGCCAGATGGTCTACGCCGACAGCCTGACGGCGATCTC    720
WP187572401.1     CTCCTGCGAAGGCGACGACTGTCGCCAGATGGTCTACGCCGACAGCCTGACGGCGATCTC    720
                  ************************************************************

AM998375.1        GGACGACGTCTTCCGCTACAGCGACGACGCCGCGCATCCCGGCTACCTGGCGGCATTCCG    780
WCH6691           GGACGACGTCTTCCGCTACAGCGACGACGCCGCGCATCCCGGCTACCTGGCGGCATTCCG    780
PA0545            GGACGACGTCTTCCGCTACAGCGACGACGCCGCGCATCCCGGCTACCTGGCGGCATTCCG    780
CP059266.1        TGACGACGTCTTCCGCTATAGCGACGACGCCGCGCATCCCGGCTACCTGGCGGCATTCCG    780
HWW-1             GGACGACGTCTTCCGCTACAGCGACGACGCCGCGCATCCCGGCTACCTGGCGGCATTCCG    780
WP187572401.1     GGACGACGTGTTCCGCTACAGCGACGACGCCGCGCATCCCGGCTACCTGGCGGCATTCCG    780
                   ******** ******** *****************************************

AM998375.1        CAACACCCTCGCACGGGTCGCAGCGCTCGACTGCGACATCCTGGTCACCCCGCACCCCTC    840
WCH6691           CAACACCCTCGCACGGGTCGCAGCGCTCGACTGCGACATCCTGGTCACCCCGCACCCCTC    840
PA0545            CAACACCCTCGCACGGGTCGCAGCGCTCGACTGCGACATCCTGGTCACCCCGCACCCCTC    840
CP059266.1        CAACACCCTCGCACGTGTGTCAGCGCTCGACTGCGACATCCTGGTCACCCCGCACCCCTC    840
HWW-1             CAACACCCTCGCACGGGTCGCAGCGCTCGACTGCGACATCCTGGTCACCCCGCACCCCTC    840
WP187572401.1     CGACACCCTCGCACGGATCGCAGGGCTCGACTGCGAGATCCTGGTCACGCCGCATCCCTC    840
                  * *************  *  *** ************ *********** ***** *****

AM998375.1        GGCCAGCGGCCTGTGGAACCGGATCGGCCCGAGGGCCGCCGCACCGCTGATGGACACCAC    900
WCH6691           GGCCAGCGGCCTGTGGAACCGGATCGGCCCGAGGGCCGCCGCACCGCTGATGGACACCAC    900
PA0545            GGCCAGCGGCCTGTGGAACCGGATCGGCCCGAGGGCCGCCGCACCGCTGATGGACACCAC    900
CP059266.1        GGCCAGCGGCCTGTGGAACCGGATCGGCCCGAGGGCCGCCGCACCGCTGATGGACACCAC    900
HWW-1             GGCCAGCGGCCTGTGGAACCGGATCGGCCCGAGGGCCGCCGCACCGCTGATGGACACCAC    900
WP187572401.1     GGCGAGCGGCCTGTGGAACCGGATCGGCCCGAGGGCCGCCGCGCCGCTGATGGACACCAG    900
                  *** ************************************** ****************
```

```
AM998375.1         CGCCTGCCGCCGCTACGCGCAGGGCGCGAGGCAGCGGCTGGAGAAGCGCCTGGCCGAGGA    960
WCH6691            CGCCTGCCGCCGCTACGCGCAGGGCGCGAGGCAGCGGCTGGAGAAGCGCCTGGCCGAGGA    960
PA0545             CGCCTGCCGCCGCTACGCGCAGGGCGCGAGGCAGCGGCTGGAGAAGCGCCTGGCCGAGGA    960
CP059266.1         CGCCTGCCGCCGCTACGCGCAGGGCGCGATGCAGCGGCTGGAGAAGCGCCTGGCCGAGGA    960
HWW-1              CGCCTGCCGCCGCTACGCGCAGGGCGCGAGGCAGCGGCTGGAGAAGCGCCTGGCCGAGGA    960
WP187572401.1      TGCCTGCCGCCGCTACGCGCAGGGCGCGATGCAGCGGCTGGAGAAGCGCCTGGCCGAGGA    960
                    **************************** ******************************

AM998375.1         AGCCGCCACCTCCCCCTCCAGCGGCGCGCGGCCTTGATGCGCATCAATGCCCCGCGCGGC    1020
WCH6691            AGCCGCCACCTCCCCCTCCAGCGGCGCGCGGCCTTGATGCGCATCAATGCCCCGCGCGGC    1020
PA0545             AGCCGCCACCTCCCCCTCCAGCGGCGCGCGGCCTTGATGCGCATCAATGCCCCGCGCGGC    1020
CP059266.1         GGCCGCCACCTCNCCCTCCGGCGGCGCGCGGCCTTGATGCGCATCAATGCCCCGCGCGGC    1020
HWW-1              AGCCGCCACCTCCCCCTCCAGCGGCGCGCGGCCGTGATGCGCATCACTGCCCCGCGCGGC    1020
WP187572401.1      AGCCGCCACCTCCCCCGCCGGCGGCGCGCGGCCTTGATGCGCATCAATGCCCCGCGCGGC    1020
                    *********** *** ** ************* ************ *************

AM998375.1         CTGCCCGGCCACGCGAAGCGCGGCTTCTGCCCCAGCTGCGGGGCGCGGCGCATGGCCGAG    1080
WCH6691            CTGCCCGGCCACGCGAAGCGCGGCTTCTGCCCCAGCTGCGGGGCGCGGCGCATGGCCGAG    1080
PA0545             CTGCCCGGCCACGCGAAGCGCGGCTTCTGCCCCAGCTGCGGGGCGCGGCGCATGGCCGAG    1080
CP059266.1         CTGCCCGGCCACGCTGGCCTCCCTTTCCCATGCGCGGCG-TGACGCTGCGCGGATCCCAC    1079
HWW-1              CTGCCCGGCCACGCTGGCCTCCCTTTCCCATGCGCGGCG-TGACGCTGCGCGGATCCCAC    1079
WP187572401.1      GTACCCCGCCACGCTGGCCTCCCTTTCCCATGCGCGGCG-TGACGCTGCGCGGATCCCAC    1079
                    * *** *******    * *   ***     *        * *** ****    ** *

AM998375.1         AGCGCGCGGC------------ACCTGGTGGAGGAGGTGTTTGGCCCGCGGCCGGTGCGG    1128
WCH6691            AGCGCGCGGC------------ACCTGGTGGAGGAGGTGTTTGGCCCGCGGCCGGTGCGG    1128
PA0545             AGCGCGCGGC------------ACCTGGTGGAGGAGGTGTTTGGCCCGCGGCCGGTGCGG    1128
CP059266.1         ACCATGAAGAAGAACCCGTCCCGCACCGCCGCCCTGCCCTTCGAGATCGTCGCCACGCGC    1139
HWW-1              ACCATGAAGAAGAACCCGTCCCGCACCGCCGCCCTGCCCTTCGAGATCGTCGCCACGCGC    1139
WP187572401.1      ACCATGAAGAAGAACCCGTCCCGCACCGCCGCCCTGCCCTTCGAGATCGTCGCCACGCGC    1139
                   * *  *  *              *   *  *    *   ** *         *   ***

AM998375.1         CAATGGGTGCTGAGTTTTCCGTACCCGTTGCGCTTCCTGTTCGCCAGCAAGCCGGAGGCC    1188
WCH6691            CAATGGGTGCTGAGTTTTCCGTACCCGTTGCGCTTCCTGTTCGCCAGCAAGCCGGAGGCC    1188
PA0545             CAATGGGTGCTGAGTTTTCCGTACCCGTTGCGCTTCCTGTTCGCCAGCAAGCCGGAGGCC    1188
CP059266.1         GAACATCCGCTGGGCATGCCGGC---CGAGCGCTTCCTGCGCGACTACTGGCACAAGCGG    1196
HWW-1              GAACATCCGCTGGGCATGCCGGC---TGAGCGCTTCCTGCGCGACTACTGGCACAAGCGG    1196
WP187572401.1      GAACATCCGCTGGGCATGCCGGC---CGAGCGCTTCCTGCGCGACTACTGGCACAAGCGG    1196
                    **     **** *  * ***        **********  ** *  *  **   **
```

```
AM998375.1         ATCGGCCCGGTGCTGGGCATCGTGCATCGT-----GT-----------------------    1220
WCH6691            ATCGGCCCGGTGCTGGGCATCGTGCATCGT-----GTGATCGCC-GGTTGGCTTGCCGAT    1242
PA0545             ATCGGCCCGGTGCTGGGCATCGTGCATCGT-----GTGATCGCC-GGTTGGCTTGCCGAT    1242
CP059266.1         CCC---CTGCTGATCAGGAACGCCTTTCCCGGCTTCGAAACGCCCGTGCAGCCGGAAGAC    1253
HWW-1              CCC---CTGCTGATCAGGAACGCCTTTCCCGGCTTCGAAACACCCGTGCAGCCGGAAGAC    1253
WP187572401.1      CCA---CTGCTGATCAGGAACGCCTTTCCCGGCTTCGAAACGCCCGTGCAGCCGGAAGAC    1253
                         * * ** *  * * **    **

AM998375.1         ------------------------------------------------------------    1220
WCH6691            CAGGCCGGCGTGCCGCGGGATACGGCGCAATGCGGTGCGGTGACCCTGATCCAGCGCTTC    1302
PA0545             CAGGCCGGCGTGCCGCGGGATACGGC----------------------------------    1268
CP059266.1         CTGGCCGGCCTGGCCTGCGAAGAGGGCGTGCTGGCGCGGATGATCAGCCTGGATCGCGCC    1313
HWW-1              CTGGCCGGCCTGGCCTGCGAAGAGGGCGTGCTGGCGCGGATGATCAGCCTGGACCGCGCC    1313
WP187572401.1      CTGGCCGGCCTGGCCTGCGAAGAGGGCGTGCTGGCGCGGATGATCAGCCTGGATCGCGCC    1313

AM998375.1         ------------------------------------------------------------    1220
WCH6691            GGCAGCGCGC---TGAATCTCAACATCCACTTCCACATGC--------------------    1339
PA0545             ------------------------------------------------------------    1268
CP059266.1         ACCGGGGCCTGGGACGTGCGCACCGGCCCGTTCCAGGAAGAGGATTTCCCCGGCCTGCCC    1373
HWW-1              AGCGGGGCCTGGGACGTGCGCACCGGCCCGTTCCAGGAAGAGGATTTCCCCGGCCTGCCC    1373
WP187572401.1      ACCGGGGCCTGGGACGTGCGCACCGGCCCGTTCCAGGAAGAGGATTTCCCCGGCCTGCCC    1373

AM998375.1         ------------------------------------------------------------    1220
WCH6691            ----------------TGTGGCTCGACGGCGTGTACGAGGAC---ACCACCGAGCGTCCG    1380
PA0545             ------------------------------------------------------------    1268
CP059266.1         GACCACGACTGGACCCTGCTGGTGCAGGACGTGGACAAGTGGGATGCCGACGTACGCGAA    1433
HWW-1              GACCACGACTGGACCCTGCTGGTGCAGGACGTGGACAAGTGGGATGCCGACGTACGCGAA    1433
WP187572401.1      GACCACGACTGGACCCTGCTGGTGCAGGACGTGGACAAGTGGGATGCCGATGTACGCGAA    1433

AM998375.1         ------------------------------------------------------------    1220
WCH6691            CAGCGCAAGC------CGCGCCTGCACCGCACC---------------------------    1407
PA0545             ------------------------------------------------------------    1268
CP059266.1         CTGCTGGCGCAGTTCCGTTTCCTGCCGCGCTGGCGGGTGGACGACATCATGATCAGCTTC    1493
HWW-1              CTGCTGGCGCAGTTCCGTTTCCTGCCGCGCTGGCGGGTGGACGACATCATGATCAGCTTC    1493
WP187572401.1      CTGCTGGCGCAGTTCCGTTTCCTGCCGCGCTGGCGGGTGGACGACATCATGATCAGCTTC    1493
```

```
AM998375.1       ------------------------------------------------------------   1220
WCH6691          ------------------------------------------------------------   1407
PA0545           ------------------------------------------------------------   1268
CP059266.1       GCGGCCACCGGCGGCTCGGTGGGCGCCCACGTGGACCACTACGACGTGTTCCTGCTGCAG   1553
HWW-1            GCGGCCACCGGTGGCTCGGTGGGCGCCCACGTGGACCACTACGACGTGTTCCTGCTGCAG   1553
WP187572401.1    GCGGCCACCGGCGGCTCGGTGGGCGCCCACGTGGACCACTACGACGTGTTCCTGCTGCAG   1553

AM998375.1       ------------------------------------------------------------   1220
WCH6691          ------------------------------------------------------------   1407
PA0545           ------------------------------------------------------------   1268
CP059266.1       GCACAGGGCGAGCGCCGCTGGATGATCGACGCCAGCGTGGCGCTGGGGAAGCCCGCACCT   1613
HWW-1            GCACAGGGCGAGCGCCGCTGGATGATCGATGCCAGCGTGGCGTTGGGGAAGCCCGCACCT   1613
WP187572401.1    GCACAGGGCGAGCGCCGCTGGATGATCGACGCCAGCGTGGCGCTCGGGAAGCCGGCGCCG   1613

AM998375.1       ------------------------------------------------------------   1220
WCH6691          ------------------------------------------------------------   1407
PA0545           ------------------------------------------------------------   1268
CP059266.1       GACCTGGCCTTCCAGGAGGACGTGGCGATCAAGCTGCTGCAGCGGTTCGCCCCGACCCAC   1673
HWW-1            GACCTGGCCTTCCAGGAGGACGTGGCGATCAAGCTGCTGCAGCGTTTCGCCCCGACCCAC   1673
WP187572401.1    GACCTAGCCTTCCAGGAGGACGTGGCGATCAAGCTGCTGCAGCGGTTCGCCCCGACCCAC   1673

AM998375.1       ------------------------------------------------------------   1220
WCH6691          ------------------------------------------------------------   1407
PA0545           ------------------------------------------------------------   1268
CP059266.1       GAATGGGTGCTGTCGCCCGGTGACATGCTGTACCTGCCGCCGCTGGTCCCGCACCATGGC   1733
HWW-1            GAATGGGTGCTGTCGCCCGGCGACATGCTGTACCTGCCGCCGCTGGTCCCGCACCATGGC   1733
WP187572401.1    GAATGGGTGCTGTCGCCCGGCGACATGCTGTACCTGCCGCCGTTGGTCCCGCACCATGGC   1733

AM998375.1       ------------------------------------------------------------   1220
WCH6691          ------------------------------------------------------------   1407
PA0545           ------------------------------------------------------------   1268
CP059266.1       GTGGCGAAGAACCCGTGCCTGACCTTCTCGGTGGGCATGCGCGCACCGTCGTCGGCGGAA   1793
HWW-1            GTGGCGGAGAACCCGTGCCTGACCTTCTCGGTGGGCATGCGCGCACCGTCGTCGGCAGAA   1793
WP187572401.1    GTGGCGGAGAACCCGTGCCTGACTTTCTCGGTGGGCATGCGCGCAC--------------   1779
```

Figure S1. The *bla*$_{\text{AIM-1}}$ genomic environment from HWW-1 wastewater sample is similar with *P. mexicana* which lacks ISCR15 compared to the *P. aeruginosa*. Nucleotide sequence alignment of closely related *bla*$_{\text{AIM-1}}$ gene in this study (*P. aeruginosa* PA0545, WCH6691

and HWW-1) and from *P. mexicana*. The genetic environments carried out by a multiple sequence alignment revealed exactly similar origin of insertion sequence (*oriIS*) of ISCR15A (highlighted as orange) and ISCR15B (golden) in *P. aeruginosa* but absent in HWW-1 and *P. mexicana* whereas all sequences have similar ribosomal binding site (RBS)(green), *bla*$_{AIM-1}$ gene sequence (blue) and downstream region of *bla*$_{AIM-1}$ gene also have near perfect sequence identity between these sequences. Dashes represent nucleotides that are lacking in the indicated sequence and asterisks indicate identical nucleotides.

```
PA0545_OprD     MKVMKWSAIALAVSAGSTQFAVADAFVSDQAEAKGFIEDSSLDLLLRNYYFNRDGKEGRG     60     Loop 1
NP_249649.1     MKVMKWSAIALAVSAGSTQFAVADAFVSDQAEAKGFIEDSSLDLLLRNYYFNRDGKSGSG     60
WCH6691_OprD    ------------------------------------------------------------     0

PA0545_OprD     DRVDWTQGFLTTYESGFTQGTVGFGVDAFGYLGLKLDGTSDKTGTGNLPVMNDGKPRDDY     120    Loop 1 end and Loop 2
NP_249649.1     DRVDWTQGFLTTYESGFTQGTVGFGVDAFGYLGLKLDGTSDKTGTGNLPVMNDGKPRDDY     120
WCH6691_OprD    ---------------------MGFGVDAFGYLGLKLDGTSDKSGTGNLPVMNDGTPRDDY     39
                                     :********************:***********.*****

PA0545_OprD     SRAGGALKVRISKTILKWGEMQPTAPVFAAGGSRLFPQTATGFQLQSSEFEGLDLEAGHF     180    Loop 3
NP_249649.1     SRAGGAVKVRISKTMLKWGEMQPTAPVFAAGGSRLFPQTATGFQLQSSEFEGLDLEAGHF     180
WCH6691_OprD    SRAGGAVKVRISKTMLKWGEMQPTAPVFAAGGSRLFPQTATGFQLQSSELEGLDLEAGHF     99
                ******:*******:**********************************:**********

PA0545_OprD     TEGKQGTTTKSRGELYATYAGQTAKSADFAGGRYAITDNLSASLYGAELKDIYRQYYLNT     240    Loop 4 and 5
NP_249649.1     TEGKEPTTVKSRGELYATYAGETAKSADFIGGRYAITDNLSASLYGAELEDIYRQYYLNS     240
WCH6691_OprD    TEGKQGTTTKSRGELYATYAGETAKSADFIGGRYAITDNLSASLYGAELEDIYRQYYLNS     159
                ****: **.************:******* *******************:*********:

PA0545_OprD     NYTIPLASDQSLGFDFNIYRTTDEGKAKAGDISNTAWSLAGAYTLDAHTFTLAYQQVHGD     300    Loop 6 and start of 7
NP_249649.1     NYTIPLASDQSLGFDFNIYRTNDEGKAKAGDISNTTWSLAAAYTLDAHTFTLAYQKVHGD     300
WCH6691_OprD    NYTIPLASDQSLGFDFNIYRTNDEGKAKAGDISNTTWSLAAAYTLDAHTFTLAYQKVHGD     219
                *********************.*************:****.**************:****

PA0545_OprD     EPFDYIGFGENGSGGGGDSIFLANSVQYSDFNGPGEKSWQARYDLNMASYGVPGLTFMVR     360    Loop 7
NP_249649.1     QPFDYIGFGRNGSGAGGDSIFLANSVQYSDFNGPGEKSWQARYDLNLASYGVPGLTFMVR     360
WCH6691_OprD    QPFDYIGFGENGSGGGGDSIFLANSVQYSDFNGPGEKSWQARYDLNLASYGVPGLTFMVR     279
                :********.****.*******************************:*************

PA0545_OprD     YINGKDIDGTKVDSSSS--YAGLYGEDGKHHETNLEAKYVVQAGPAKDLSFRIRQAWHRA     418    Loop 8
NP_249649.1     YINGKDIDGTKMSDNNVGYKNYGYGEDGKHHETNLEAKYVVQSGPAKDLSFRIRQAWHRA     420
WCH6691_OprD    YINGKDIDGTKMSDNNVGYKNYGYGEDGKHHETNLEAKYVVQSGPAKDLSFRIRQAWHRA     339
                ***********:....       *******************:*****************

PA0545_OprD     NADEGEGDQNEFRLIVDYPLSIL    441                                          Loop 9
NP_249649.1     NADQGEGDQNEFRLIVDYPLSIL    443
WCH6691_OprD    NADQAEGDQNEFRLIVDYPLSIL    362
                ***:.******************
```

Figure S2. Alignment of the OprD variants identified among carbapenem resistant WCH6691 and PA0545 *P. aeruginosa* isolates as compared to the *P. aeruginosa* PAO1 (Accession number: NP_249649.1). Sequence variants from PAO1 are labeled in the bottom as a: (colon), a. (period) or a space for mismatch, each loop is highlighted with yellow.

Table S3. AIM-1 gene sample source and geographic location

Description	Query Cover	Per. ident	Accession	Place	Organism source	Year of isolation	Strain
subclass B3 metallo-beta-lactamase AIM-1 [Pseudomonas aeruginosa]	100%	100	WP_063857820.1	Australia, Adelaide	Wound	2006	WCH2677
SubclassB3 [uncultured bacterium]	100%	98.68	AMP57240.1	Latin American, El Salvador	Soil	2013	None
AIM family subclass B3 metallo-beta-lactamase [Pseudoxanthomonas mexicana]	100%	97.69	WP_162108801.1	Mexico	Anaerobic digester	2004	DSM17121
AIM family subclass B3 metallo-beta-lactamase [Pseudoxanthomonas mexicana]	100%	96.37	WP_185895986.1	China	Wastewater	2015	GTZY
AIM family subclass B3 metallo-beta-lactamase [Pseudoxanthomonas mexicana]	100%	96.04	WP_187572401.1	China	Wastewater	2015	GTZY2
Pseudoxanthomonas sp. SSD1	100%	96.37	QLQ29376.1	Singapore	activated sludge	2018	Pseudoxanthomonas sp. isolate SSD1 chromosome
SubclassB3 [uncultured bacterium]	100%	96.37	AMP57086.1	Latin American, El Salvador	Soil	2013	None
hypothetical protein A2213_09170 [Xanthomonadales bacterium RIFOXYA1_FULL_68_6]	100%	91.75	OHE90088.1	United States, Colorado, Rifle	Groundwater	2012	Xanthomonadales bacterium RIFOXYA1
MULTISPECIES: subclass B3 metallo-beta-lactamase [unclassified Pseudoxanthomonas]	97%	78.38	WP_093489397.1	United States	Plant root	Submitted 2016	Pseudoxanthomonas sp. YR558
Metallo-beta-lactamase class B [Pseudoxanthomonas sp. YR558]	97%	78.38	SFV26788.1	United States	Plant root	Submitted 2016	Pseudoxanthomonas sp. YR558
subclass B3 metallo-beta-lactamase [Pseudoxanthomonas sp. Root65]	97%	82.03	WP_055943502.1	Germany	Plant root	2012	Pseudoxanthomonas sp. Root65

Subclass B3 metallo-beta-lactamase [Pseudoxanthomonas sp. PXM02]	97%	79.12	WP_192310809.1	USA	Soil	2019	Pseudoxanthomonas sp. PXM02
Subclass B3 metallo-beta-lactamase [Pseudoxanthomonas sp. PXM01]	93%	81.63	WP_192271228.1	USA	Soil	2019	Pseudoxanthomonas sp. PXM01
Subclass B3 metallo-beta-lactamase [Pseudoxanthomonas sp. 3HH-4]	91%	83.81	WP_142125325.1	USA	Plant	Submitted 2019	Pseudoxanthomonas sp. 3HH-4
Subclass B3 metallo-beta-lactamase [Pseudoxanthomonas sp. PXM03]	90%	83.15	WP_192201055.1	USA	Soil	2018	Pseudoxanthomonas sp. PXM03
subclass B3 metallo-beta-lactamase [Pseudoxanthomonas mexicana]	86%	85.44	WP_062355367.1	USA, Ohio	hospital shower hose biofilm	2012	Pseudoxanthomonas mexicana
Subclass B3 metallo-beta-lactamase [Pseudoxanthomonas japonensis]	98%	81.14	WP_162338672.1	Japan	Urban riverside soil	2004	Pseudoxanthomonas japonensis strain:DSM 17109
Subclass B3 metallo-beta-lactamase [Xanthomonadaceae bacterium]	98%	80.47	NCT71185.1	China	contaminated soil	2018	Xanthomonadaceae bacterium
Subclass B3 metallo-beta-lactamase [Xanthomonas sp.]	86%	84.29	MBA3929446.1	USA	drinking water system	2014	Xanthomonas sp.
Subclass B3 metallo-beta-lactamase [Pseudoxanthomonas sp. CF385]	90%	81.68	WP_093297772.1	none	None	submitted 2016	Pseudoxanthomonas sp. CF385
Hypothetical protein ASD72_09730 [Pseudoxanthomonas sp. Root630]	86%	82.38	KRA44287.1	Germany	Plant root	2013	Pseudoxanthomonas sp. Root630
Subclass B3 metallo-beta-lactamase [Pseudoxanthomonas sp. Root630]	86%	82.38	WP_081420358.1	Germany	Plant root	2013	Pseudoxanthomonas sp. Root630

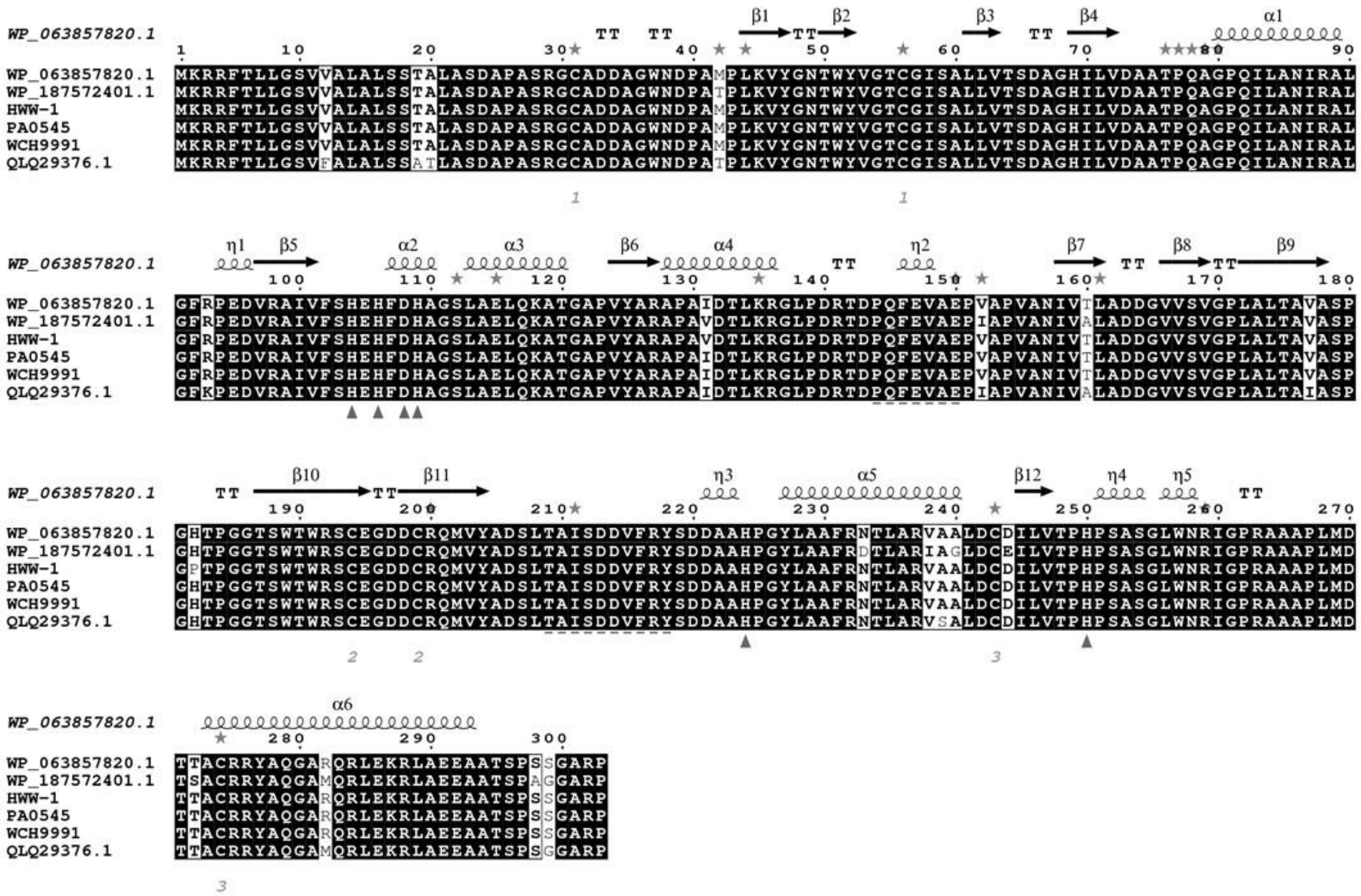

Figure S3. The amino acid sequence comparison of AIM-1 from *P. aeruginosa* and *P. mexicana* revealed conserved amino acid residues for metal binding in *bla*$_{AIM-1}$ gene. The sequences are referred by their accession number or isolate number followed by source of organism. Difference in the amino acid sequences are noted by a single letter representing the amino acid change within a particular sequence. The residues acting as zinc binding (triangle), the loop region important for substrate interaction (dashed line) and conserved residues (numbered 1-3) are indicated and were conserved in all sequences.
